# Peglated-H1/pHGFK1 nanoparticles enhance anti-tumor effects of sorafenib by inhibition of drug-induced autophagy and stemness in renal cell carcinoma

**DOI:** 10.1186/s13046-019-1348-z

**Published:** 2019-08-19

**Authors:** Xiaoge Gao, Pin Jiang, Qian Zhang, Qian Liu, Shuangshuang Jiang, Ling Liu, Maomao Guo, Qian Cheng, Junnian Zheng, Hong Yao

**Affiliations:** 1 Cancer Institute, Xuzhou Medical University, Xuzhou, Jiangsu Province 221002 People’s Republic of China; 2 Center of Clinical Oncology, Affiliated Hospital of Xuzhou Medical University, Xuzhou, Jiangsu Province 221002 People’s Republic of China; 3 Department of Cancer Biotherapy Center, Third Affiliated Hospital of Kunming Medical University, Kunming, Yunnan Province 650118 People’s Republic of China

**Keywords:** HGFK1, Sorafenib, Renal cell carcinoma, Autophagy, Cancer stem cell

## Abstract

**Background:**

Tumor targeting small molecular inhibitors are the most popular treatments for many malignant diseases, including cancer. However, the lower clinical response and drug resistance still limit their clinical efficacies. HGFK1, the first kringle domain of hepatocyte growth factor, has been defined as a potent anti-angiogenic factor. Here, we aimed to develop and identify novel nanoparticles—PH1/pHGFK1 as potential therapeutic agents for the treatment of renal cell carcinoma (RCC).

**Methods:**

We produced a novel cationic polymer—PH1 and investigated the anti-tumor activity of PH1/pHGFK1 nanoparticle alone and its combination therapy with sorafenib in RCC cell line xenografted mice model. Then, we figured out its molecular mechanisms in human RCC cell lines in vitro.

**Results:**

We firstly demonstrated that intravenous injection of PH1/pHGFK1 nanoparticles significantly inhibited tumor growth and prolonged the survival time of tumor-bearing mice, as well as synergistically enhanced anti-tumor activities of sorafenib. Furthermore, we elucidated that recombinant HGFK1 improved sorafenib-induced cell apoptosis and arrested cell cycle. In addition, HGFK1 could also decrease sorafenib-induced autophagy and stemness via blockading NF-κB signaling pathway in RCC both in vitro and in vivo.

**Conclusions:**

HGFK1 could inhibit tumor growth, synergistically enhance anti-tumor activities of sorafenib and reverse its drug resistance evolution in RCC. Our results provide rational basis for clinical application of sorafenib and HGFK1 combination therapy in RCC patients.

**Electronic supplementary material:**

The online version of this article (10.1186/s13046-019-1348-z) contains supplementary material, which is available to authorized users.

## Background

Renal cell carcinoma (RCC), also known as renal adenocarcinoma, originates in the lining of the proximal convoluted tubule and accounts for 90% of renal malignancies with increasing incidence [1]. 25–30% of RCC patients originally diagnose with metastatic diseases [2]. Radical nephrectomy is the first line treatment for patients with localized RCC, but approximately 30% of them will relapse. The median survival of patients with metastasis RCC is only 13 months [2]. Due to RCC being insensitive to radiotherapy or chemotherapy, its therapeutic intervention greatly relied on immunotherapy using cytokines, such as interleukin-2 and interferon-α, but that has been moved towards molecular targeted therapies in this decade [3, 4].

As one of the most vascular of solid cancers, angiogenesis is essential for the development of RCC [5, 6]. In recent years, a couple of tyrosine-kinase inhibitors with anti-angiogenesis ability have been approved to be as the standard cares for the treatment of metastatic RCC by the United States Food and Drug Administration (USFDA) due to the enhanced therapeutic efficacies and reduced toxicity response as compared with immunotherapy [4]. Among these, sorafenib could effectively block angiogenesis, inhibit proliferation and induce apoptosis of cancer cell as well as exhibited the most therapeutic efficacies for patients with metastasis RCC [7]. However, its clinical outcomes are still limited by serious side effects caused by long-term and high-dose exposure and rapidly developed drug resistance [8]. For the reason, combination therapies between sorafenib and other oncogenic pathway inhibitors have been the alternative options, recently [9, 10]. However, combination of two cytotoxic compounds also led to additional toxic responses that caused poor tolerance of patients with metastasis RCC [11]. Particularly, sorafenib induced drug resistance of RCC remains to be overcome [12]. Thus, there is an urgent need for an agent to be safer and more effective combination treatment of sorafenib.

Recent studies have connected sorafenib-induced drug resistance with sorafenib-induced autophagy and drug-enhanced cancer stemness properties in hepatocellular carcinoma (HCC), another USFDA-approved indication [13–16]. It is conceivable that cancer stemness properties of RCC are also associated with sorafenib-induced therapeutic resistance. Kringle 1 domain of human hepatocyte growth factor (HGFK1) belongs to hepatocyte growth factor-α (HGF-α) chain and contains a high-affinity binding domain of MET [17, 18], which has been previously defined as a potent anti-angiogenic factor [19], and over-expression of HGFK1 also showed anti-tumor activities in breast cancer cells [20], colon cancer cells [21] and HCC [22, 23]. Our more recent works showed that a nanoparticle-mediated plasmid encoding HGFK1 gene (H1/pHGFK1) exerts anti-tumor and radiosensitive activities through inhibiting MET signaling pathway, an important cancer cells “stemness” regulator in glioblastoma [10, 24]. Accordingly, we characterized the anti-tumoral activities of HGFK1 and its synergistic efficacies with sorafenib to RCC in the present study.

Aiming to effectively export the activity of HGFK1 in vivo, gene therapy is our optimal approach in current study. Previously, we have developed a cationic co-polymer consisting of low molecular weight polythylenimine (PEI, 600 Da) linked by β-cyclodextrin and conjugated with folic acid (named H1) [25, 26]. As a gene delivery vector, H1-formed nanoparticle exhibits effective gene delivery and low cytotoxicity via intratumoral injection [24, 26]. However, our recent data showed that it was easy to be blocked by reticuloendothelial system (RES) of body in case of systemic administration (data not show). Polyethylene glycol (PEG), a neutral and hydrophilic polymer, is often used to modify nanoparticles in order to avoid the clearance by RES, which results in prolonging the circulation time of polyplexes [27].

In this study, we produced a novel cationic polymer, Peglated-H1 (PH1), via mixing H1 with the PEG (M.W. 3350)-grafted PEI-CyD. We firstly characterized the physical properties of PH1/plasmid nanoparticles and their tissue distribution by using tail vein injection. And then, we investigated the anti-tumor activity of PH1/pHGFK1 nanoparticles alone and the combination therapy of PH1/pHGFK1 and sorafenib in a human RCC cell line xenografted mouse model. Finally, we figured out the molecular mechanism of HGFK1 synergistically enhancing the anti-tumor efficacies of sorafenib.

## Methods

### Cell culture and antibodies

RCC cell lines Ketr-3, 786-O, and ACHN as well as normal renal tubular epithelial cell HK-2 were all cultured in DMEM (high glucose) medium supplemented with 10% fetal bovine serum (FBS), 100 IU/ml penicillin and streptomycin. All the cultures were maintained in humidified incubator with 5% CO_2_ at 37 °C. The antibodies against β-actin, CD133, Nanog, Oct4, GAPDH, phosphorylated (Serine-311) and total NF-κB p65, PARP were obtained from Cell Signaling Technology (Danvers, MA, USA). The antibody against LC3B was purchased from Novus (Shanghai, China), and Ki-67 was purchased from Abcam (Shanghai, China). All the secondary antibodies labeled with HRP were purchased from Beyotime (Nanjing, China). B-27 supplement (B-27) was purchased from Thermo Fisher Scientific (Shanghai, China). Epidermal growth factor (EGF) and basic fibroblast growth factor (bFGF) were purchased from PeproTech (Rocky Hill, NJ, USA). Insulin was purchased from Sigma (Shanghai, China). Bay 11–7082 (inhibitor of NF-κB) was purchased from MCE (Shanghai, China). Sorafenib tosylate tablets were purchased from Bayer (Shanghai, China). 3-Methyladenine (3-MA, an inhibitor of PI3K) was purchased from TargetMol (Shanghai, China). YOYO-1 dye was purchased from Invitrogen (Carlsbad, CA, USA).

### Preparation of recombinant HGFK1 protein and HGFK1 eukaryotic expression plasmid

The gene fragment encoding the entire HGFK1 was cloned in prokaryotic expression vector pTXB1 and expressed in *E. coli* BL21 (DE3) through inducement by IPTG. The fusion protein containing recombinant HGFK1 (rHGFK1) and intein (chitin binding domain) tag were purified using chitin affinity beads and then cleaved using DTT according to the manufacture’s instruction. The purity and concentration of rHGFK1 were respectively analyzed with SDS-PAGE and a BCA protein concentration kit (Beyotime, Nanjing, China).

The cDNA fragment encoding IgK leader and HGFK1 was constructed into eukaryotic expression vector pORF-Luc (Invitrogen, Carlsbad, CA, USA) to generate pORF-HGFK1 plasmid (pHGFK1). All the plasmids were purified with a PureLink™ Hipure plasmid maxiprep kit (Invitrogen, Carlsbad, CA, USA).

### Cell proliferation assay

The effects of sorafenib and HGFK1 on cell proliferation were measured with a CCK-8 assay kit (VICMED, Xuzhou, China). The cells were seeded on 96-well plates at a density of 5, 000 cells per well in 100 μl culture medium and allowed to adhere overnight. Subsequently, the cells were incubated with sorafenib and/or rHGFK1 at the increasing concentrations dissolved in DMEM medium supplemented with 2% FBS for 48 h. The CCK-8 dye was added and incubated for further 2 h. The absorbance was finally determined at 450 nm using a microplate reader (Bio-Tek Instruments, Winooski, USA).

### Cell cycle assay

The RCC cells were seeded on 6-well plates and cultured overnight. Then, the cells were treated with sorafenib and/or rHGFK1 for 48 h at the indicated concentrations. After typsinized, washed, and fixed, the cells were incubated with 100 mg/ml RNase A and stained with PI at 37 °C for 30 min in the dark. Finally, the cells were examined on a flow cytometer (BD Biosciences, USA). More than 1 × 10^5^ cells were analyzed for each measurement.

### Cell apoptosis assay

The cultured RCC cells were treated with sorafenib and/or rHGFK1 at the indicated concentrations for 48 h, and then stained with an Annexin V-FITC/PI apoptosis detection kit (KeyGen, Nanjing, China) according to the manufactured instructions. Finally, flow cytometer was used to detect cellular apoptosis. More than 1 × 10^6^ cells were analyzed for each measurement.

### SDS-PAGE and Western-blotting assay

RCC cells treated with sorafenib and/or rHGFK1 were lysed in RIPA buffer with protease inhibitor on ice for 30 min. The supernatant was collected after centrifuging at 13, 000 g for 15 min, and protein content was quantified with a BCA protein concentration kit (Beyotime, Shanghai, China). And then, equal quantities of protein samples were separated with SDS-PAGE and transferred to PVDF membrane. The membrane was blocked with 5% nonfat-dried milk for 1 h at room temperature. Primary antibodies were added and incubated overnight at 4 °C. The next day, secondary antibodies were added and incubated for 1 h at room temperature. Chemical signals were visualized with Tanon imaging system (Tanon Science & Technology Co., Ltd., China).

### mRFP-GFP-LC3 puncta formation assay

RCC cells were plated on the coverslips and infected with mRFP-GFP-LC3 lentivirus purchased from Hambio (Wuhan, China). After treated with sorafenib and/or rHGFK1, autophagy flux was detected via observing the red, green, and yellow fluorescent puncta formation under fluorescence microscopy. RFP fluorescence is stable in acidic conditions, but GFP fluorescence is sensitive to acidic environments. Thus, co-localization of RFP and GFP fluorescence (corresponding to yellow) indicates autophagosomes, and single red fluorescence indicates autolysosomes, because GFP fluorescence is quenched in low lysosomal pH.

### Mammosphere formation assay

The mammosphere formation assay was performed according to the previously reported protocol [28]. Firstly, RCC cells were pre-treated with sorafenib and/or rHGFK1 for 48 h, and then the viable cells were collected and re-suspended in serum-free DMEM/F12 medium supplemented with EGF, bFGF, insulin, B-27, and BSA. Finally, 2000 cells were seeded on each well of the ultralow attachment 6-well plates (Corning Costar, Corning, NY, USA). After 2 weeks, the primary mammospheres were collected and enzymatically dissolved. Then, 2000 cells were seeded on the ultralow attachment 6-well plates again. Two weeks later, the mammospheres were observed and analyzed under microscopy (Olympus Life Science, Tokoyo, Japan).

### Synthesis and characterization of PH1

Firstly, PEI_600_-CyD backbone was synthesized according to the method reported previously [26]. PEG with molecular weight of 3350 Da (PEG_3350_, 250 mg) dissolved in DMSO was stirred until the mixture solution was clear. 1, 10-carbon-yldiimidazole (CDI, 40 mg) was added under nitrogen atmosphere and stirred for further 4 h. Then, the mixture was added to PBS solution of PEI_600_-CyD (240 mg) and stirred overnight. The crude product of PEG_3350_-PEI_600_-CyD was purified by dialysis in water for 2 days and then used to obtain PEG_3350_-PEI_600_-CyD powder. 1 mg of PEG_3350_-PEI_600_-CyD dissolved in 0.5 ml D_2_O was analyzed by using H^1^ NMR (ECS400, JEOL, Japan). H1 and PEG_3350_-PEI_600_-CyD were mixed at the molar ratio of 1:1 to produce Peglated-H1 (named PH1).

### Preparation and characterization of PH1/plasmid nanoparticles

For in vitro transfection efficiency assay, PH1 was added to the plasmid solution at different N/P ratios (where ‘N’ is the amount of nitrogen in PEI and ‘P’ is the amount of phosphate in 1 μg of DNA) according to previously reported [26]. Then, PH1/plasmid nanoparticles were allowed to incubate for 20 min at room temperature prior to transfection. The particle size and zeta potential of nanoparticles were measured by a Zeta potential/Particle sizing systems (Nicomp 380 ZLS, USA).

For in vivo treatment, PH1 was mixed with pHGFK1 dissolved in 5% sucrose solution at the indicated N/P ratios. Then, the PH1/pHGFK1 nanoparticles were freeze-dried and re-suspended in deionized water prior to injection.

For in vivo distribution assay, pHGFK1 was stained with fluorescent dye YOYO-1 according to previously reported [29]. Then, PH1/YOYO-1-pHGFK1 nanoparticles were prepared and administrated by intravenous injection. The fluorescent signal was captured by the in vivo optical imaging systems (Berthold Technologies, Germany). In order to analyze the expression of PH1 loaded plasmids in vivo, the tumor-bearing mice were given PH1 loaded control plasmids (PH1/pVehicle, which can express GFP protein) by intravenous injection. The GFP fluorescence was monitored via in vivo optical imaging systems at the indicated time points.

### Xenograft tumor

Fifty female Balb/c nude mice (4–6 weeks old) were purchased from Beijing Vital River Laboratory Animal Technology Co., Ltd. (Beijing, China) and maintained at Xuzhou Medical University Animal Center (Xuzhou, China). The mouse was subcutaneously inoculated in the flank with Ketr-3 cells (1 × 10^7^ cells). After one week, the mice with comparable tumor size were randomly divided into 5 groups. For the sorafenib group, the mice were given 50 mg/kg sorafenib in PBS by gavage every other day. For the PH1/pVehicle group, the mice were given 25 μg pORF-Luc vectors mixed with PH1 by intravenous injection once a week. For the PH1/pHGFK1 group, the mice were given 25 μg pHGFK1 plasmids mixed with PH1 by intravenous injection once a week. For the combination group, the mice were simultaneously given pHGFK1 plasmids and sorafenib under the same conditions. For the control group, the mice received equal volume of PBS by gavage every other day. The length and width of the tumors were measured with a caliper twice a week, and tumor volumes were calculated as length×width^2^/2. Four tumor-bearing mice from each group were sacrificed and the tumors were fixed with 10% neutral formalin buffering solution. In addition, the heart, liver, spleen, lung, and kidney were also collected and fixed for further use. The remaining mice in each group were maintained for the survival study.

### Immunohistochemistry (IHC) and HE staining

The tumor was removed from xenograft nude mouse and fixed with 10% neutral formalin buffering solution, and then embedded in paraffin and sectioned into 4 μm sections. After deparaffinized, rehydrated, and boiled in 10 mM sodium citrate (pH 6.0) for 15 min, 3% H_2_O_2_ was used to block endogenous peroxides activity. After blocking nonspecific antigen, the tumor sections were incubated with antibodies against CD133, Nanog, Oct4, LC3B, and phosphorylated NF-κB overnight at 4 °C, respectively. Following incubated with HRP-labeled secondary antibody at room temperature for 30 min, the tumor sections were stained by DAB. Tissue sections were observed under a microscope, and at least three to five fields per section were analyzed and positively stained cells were counted using Image-Pro Plus 7.0 (Media Cybernetics, Inc., USA).

For HE staining, the fixed mouse internal organs including heart, liver, spleen, lung, and kidney were also sectioned into 4 μm and stained using an hematoxylin and eosin staining kit (Beyotime, Shanghai, China) according to the manufacture protocols. Tissue sections were observed under a microscope, and at least three fields per section were analyzed.

### Statistical analysis

The in vitro experiments were repeated at least three times and the results were presented as mean ± standard deviation (SD). Two-tailed student’s *t* test was performed to determine the statistical significance of differences between groups. Survival analysis of tumor-bearing mice was performed with Kaplan-Meier method. All the data was analyzed with GraphPad Prism 5.0 (GraphPad Software, Inc., USA), and *p* < 0.05 was considered as significant difference.

## Results

### Synthesis and characterization of PH1

**Fig. 1:**
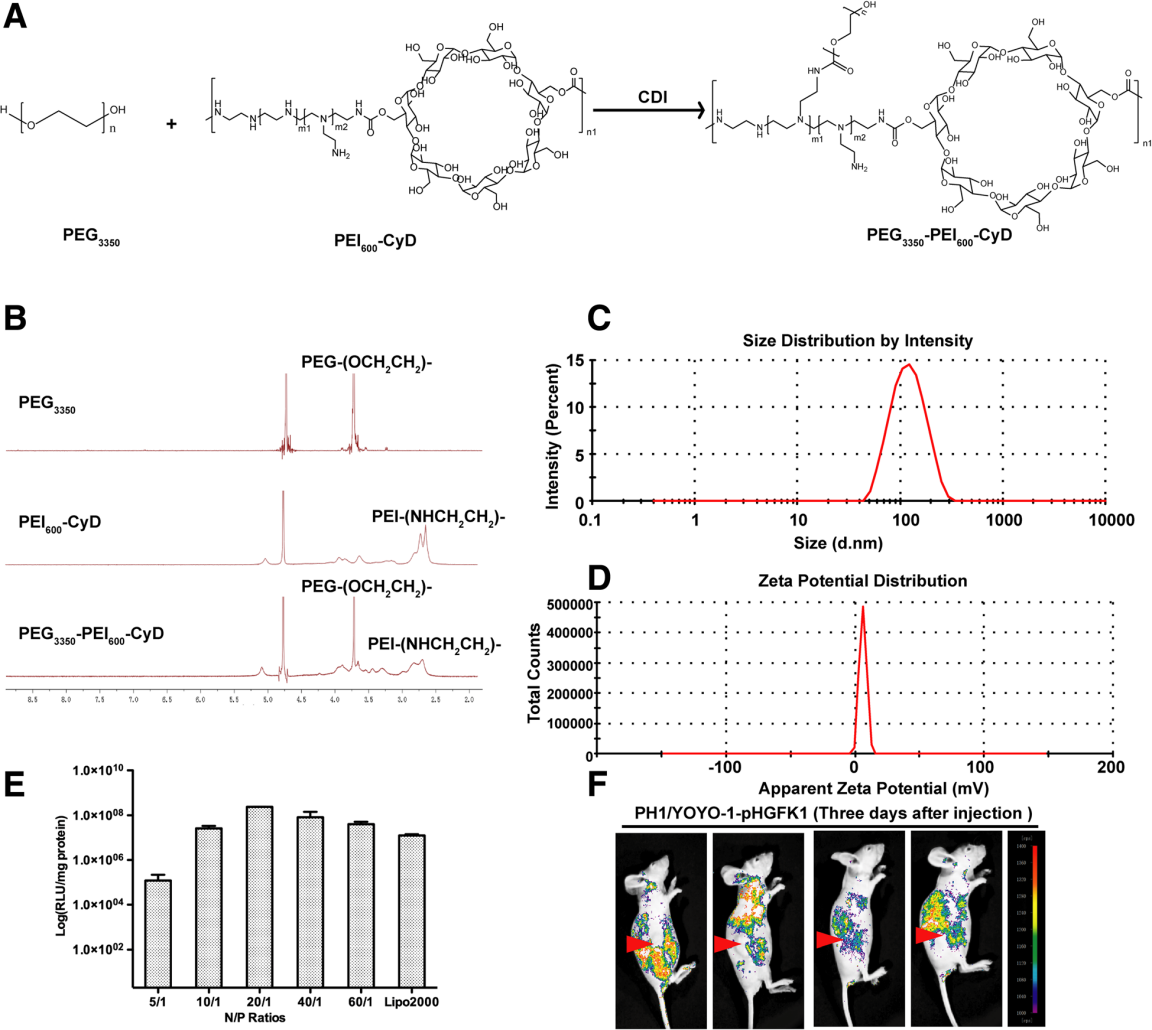
Synthesis and characterization of PH1. **a** Synthesis of PEG_3350_-PEI_600_-CyD. **b** H^1^NMR analysis of PEG_3350_, PEI_600_-CyD, and PEG_3350_-PEI_600_-CyD. **c** Particle size of PH1/plasmid polyplexes at the N/P ratio of 20/1. **d** Zeta potential of PH1/plasmid polyplexes at the N/P ratio of 20/1. **e** In vitro transfection effciency assay of PH1 in 293 T cells. **f** Distribution analysis of YOYO-1 labeled pHGFK1 was performed with in vivo optical imaging system

In our present study, PEG_3350_ was conjugated to PEI_600_-CyD polymeric backbone to afford PEG_3350_-PEI_600_-CyD (Fig. 1a). The successful conjugation of PEG to PEI-CyD was further confirmed with H^1^ NMR, our results showed that the specific signals of PEG_3350_ and PEI_600_ appeared in final products (Fig. 1b). PH1 was obtained via mixing H1 and PEG_3350_-PEI_600_-CyD at the molar ratio of 1:1. The particle size of PH1/plasmid is 106.67 ± 13.3 nm that is similar with H1/plasmid at the same N/P ratio of 20/1 (Fig. 1c). And the zeta potential of PH1/plasmid is 6.68 ± 0.33 mV at the N/P ratio of 20/1 (Fig. 1d).

The transfection efficiency of PH1 in vitro was assessed using luciferase activity assay. 293T cells were transfected with PH1/plasmid at different N/P ratios, and lipofectamine 2000 (Lipo2000) was used as positive control. As shown in Fig. 1e, the N/P ratio of 20/1 exhibited the highest luciferase activity. The in vivo optical imaging assay showed that YOYO-1 labeled plasmids (YOYO-1-pHGFK1) presented wide distribution and were partially located at tumor (red arrow) regions at the third day (Fig. 1f). In addition, significant GFP fluorescence was observed after being injected for 1 day. The strongest fluorescence was caught at the third day, and then almost disappeared at the seventh day (Additional file 1: Figure S1).

### Combination of HGFK1 and sorafenib inhibits xenograft tumor growth

**Fig. 2:**
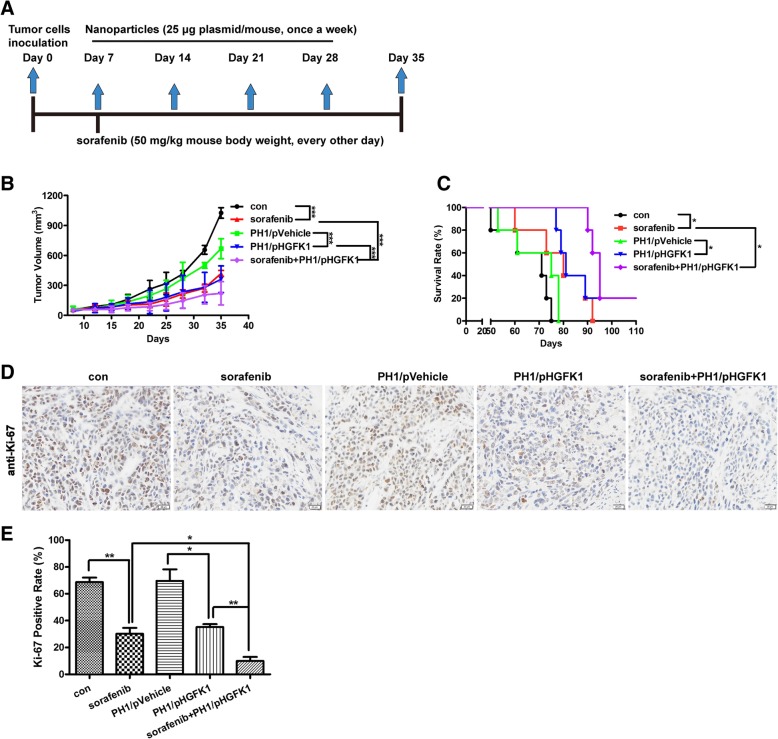
Effect of sorafenib and HGFK1 on tumor growth in vivo. Tumor-bearing mouse model was subcutaneously established by implantation of Ketr-3 cells in nude Blab/c mouse. **a** Schematic diagram illustrated the therapeutic schedule according to the *materials and methods*. **b** Tumor volume was measured every 3 days, and all data shown represent the mean ± SD from ten independent mice. **c** The survival curve of tumor-bearing mice was analyzed with the Log-rank (Mantel-Cox) test. **d** Ki-67 was stained with IHC. **e** The expression of Ki-67 was analyzed and quantified with IOD (Integrated Option Density), and all data shown represent the mean ± SD from four independent mice. Significant differences are denoted by * for *p* < 0.05, ** for *p* < 0.01, and *** for *p* < 0.001

To investigate the anti-tumor activity of HGFK1 and its synergistic roles with sorafenib in vivo, we successfully established subcutaneous xenograft tumor mouse model using Ketr-3 cells. The nanoparticles of PH1 and plasmids were prepared as described in *materials* and *methods*. After treatment for 28 days (Fig. 2a), PH1/pHGFK1 could significantly inhibit tumor growth compared to PH1/pVehicle. Furthermore, combination treatment of PH1/pHGFK1 and sorafenib exhibited stronger inhibitory activity compared to individual sorafenib or PH1/pHGFK1 treatment (Fig. 2b). Moreover, treatment of PH1/pHGFK1 or combination with sorfenib prolonged the survival time of tumor-bearing mice compared to the controls with significant difference (Fig. 2c). To explore the cytotoxity after treatment with sorafenib or PH1/pHGFK1, we monitored the mouse body weight and detected the mouse internal organs including heart, liver, spleen, lung, and kidney via HE staining. Our results showed that all treatments neither affected mouse body weight nor caused obvious organ damage compared to the controls (Additional file 1: Figure S2 and S3).

Ki-67, a DNA-binding nucleus antigen, exclusively presents in proliferating cells, and is also an excellent marker to measure tumor cell proliferation [30]. Our IHC results showed that PH1/pHGFK1 treatment could significantly decrease Ki-67 positive cell ratio (35.20%) compared to PH1/pVehicle treatment (69.60%). In addition, combination treatment of PH1/pHGFK1 and sorafenib exhibited the lowest Ki-67 positive cell ratio (10%) compared to either sorafenib (30.20%) or PH1/pHGFK1 treatment (Fig. 2d and e). Altogether, HGFK1 could inhibit tumor growth of RCC and synergistically enhance anti-tumor activities of sorafenib in vivo.

### Combination of HGFK1 and sorafenib inhibits cell proliferation of RCC

**Fig. 3:**
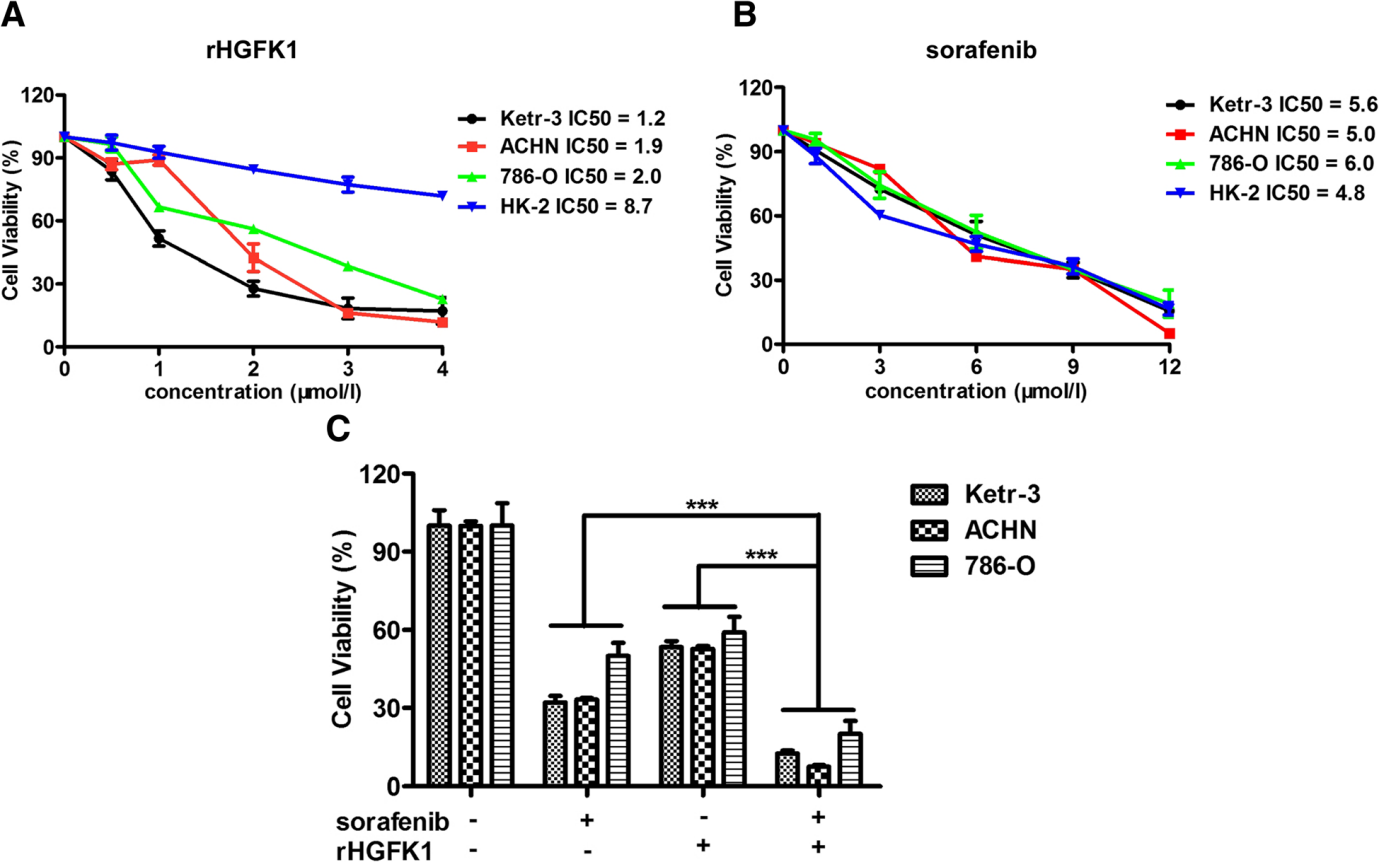
Effect of sorafenib and HGFK1 on proliferation of RCC cell lines Ketr-3, ACHN, 786-O, and normal renal tubular epithelial cells HK-2. Cell viability was determined with a CCK-8 assay Kit. **a** The cells were treated with rHGFK1 for 48 h at increasing concentrations (0, 0.5, 1, 2, 3, 4 μmol/l). rHGFK1 inhibited Ketr-3, ACHN, 786-O, and HK-2 cell growth with an IC50 of 1.2, 1.9, 2.0, and 8.7 μmol/l, respectively. **b** The cells were treated with sorafenib for 48 h at increasing concentrations (0, 1, 3, 6, 9, 12 μmol/l). Sorafenib inhibited Ketr-3, ACHN, 786-O, and HK-2 cell growth with an IC50 of 5.6, 5.0, 6.0, and 4.8 μmol/l, respectively. **c** Ketr-3 and ACHN cells were treated with sorafenib (6.0 μmol/l) and/or rHGFK1 (2.0 μmol/l) for 48 h. The IC50 values of each cell were calculated from dose-response curves using Graphpad prism 5.0. All data shown represent the mean ± SD from at least three independent experiments. Significant differences are denoted by *** for *p* < 0.001

In order to further examine the effects of HGFK1 and sorafenib on cell proliferation of RCC cell lines in vitro, we prepared the purified recombinant HGFK1 (rHGFK1) proteins from *E. coli* BL21 (DE3) (Additional file 1: Figure S4). RCC cell lines including Ketr-3, ACHN, and 786-O as well as normal renal tubular epithelial cell HK-2 were treated with rHGFK1 and/or sorafenib under the indicated concentrations for 48 h, then the cell viability was analyzed using a CCK-8 assay Kit. Our results showed that rHGFK1 could inhibit the proliferation of Ketr-3, ACHN, and 786-O cell lines with an IC50 of 1.2, 1.9, and 2.0 μmol/l, but had less effect on HK-2 cells (Fig. 3a). Interestingly, sorafenib could not only inhibit the proliferation of Ketr-3, ACHN, and 786-O cells, but also HK-2 cells in a dose-dependent manner with similar IC50 of 5.6, 5.0, 6.0, and 4.8 μmol/l, respectively (Fig. 3b). Moreover, combination of rHGFK1 (2.0 μmol/l) and sofafenib (6.0 μmol/l) exhibited stronger inhibition on cell proliferation than single rHGFK1 or sorafenib with significant difference on RCC cell lines (Fig. 3c). Taken together, HGFK1 also exhibited anti-tumor activity on RCC in vitro, and showed synergistic effect on tumor cell proliferation.

### HGFK1 improves sorafenib-induced cell apoptosis in RCC

**Fig. 4:**
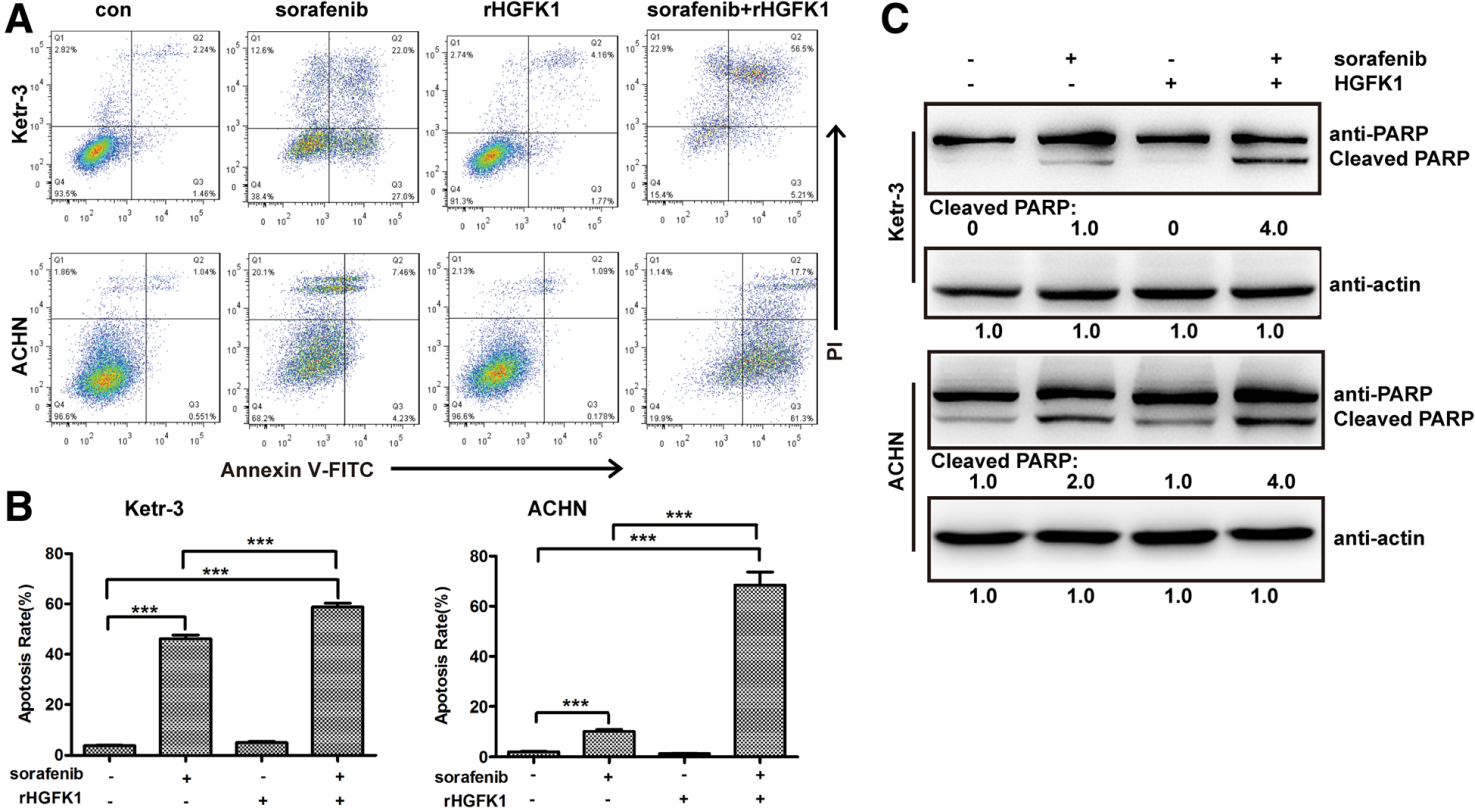
Effect of sorafenib and HGFK1 on cell apoptosis of RCC. **a** Ketr-3 and ACHN cells were treated with sorafenib and/or rHGFK1 for 48 h and stained with an Annexin V-FITC/PI apoptosis kit, and then analyzed with a flow cytometer. **b** Quantification of cell apoptosis is shown for Ketr-3 and ACHN induced by sorafenib and/or rHGFK1. **c** Western-Blotting was used to detect the cleavage of PARP induced by sorafenib and/or rHGFK1 in Ketr-3 and ACHN cells. All data shown represent the mean ± SD from at least three independent experiments. Significant differences are denoted by *** for *p* < 0.001

To elucidate the synergistic anti-proliferation mechanisms of sorafenib and HGFK1, Ketr-3 and ACHN cells were treated with sorafenib and/or rHGFK1, and then analyzed with an Annexin-V-FITC/PI apoptosis assay kit. As shown in Fig. 4a and b, compared to the control, sorafenib treatment induced 46.20 and 10.22% apoptosis on Ketr-3 and ACHN cells, respectively. However, rHGFK1 treatment did not induce obvious apoptosis on Ketr-3 and ACHN cells. Interestingly, combination treatment of rHGFK1 and sorafenib induce 58.80 and 68.40% apoptosis on Ketr-3 and ACHN cells, respectively. In addition, Western-Blotting results showed that sorafenib treatment induced the significant cleavage of PARP, but not rHGFK1. Combination of sorafenib and rHGFK1 induced more cleavage of PARP in both Ketr-3 and ACHN cells (Fig. 4c). All these results suggested that HGFK1 could promote sorafenib-induced cell apoptosis.

### HGFK1 not sorafenib induces cell cycle arrest in RCC

**Fig. 5:**
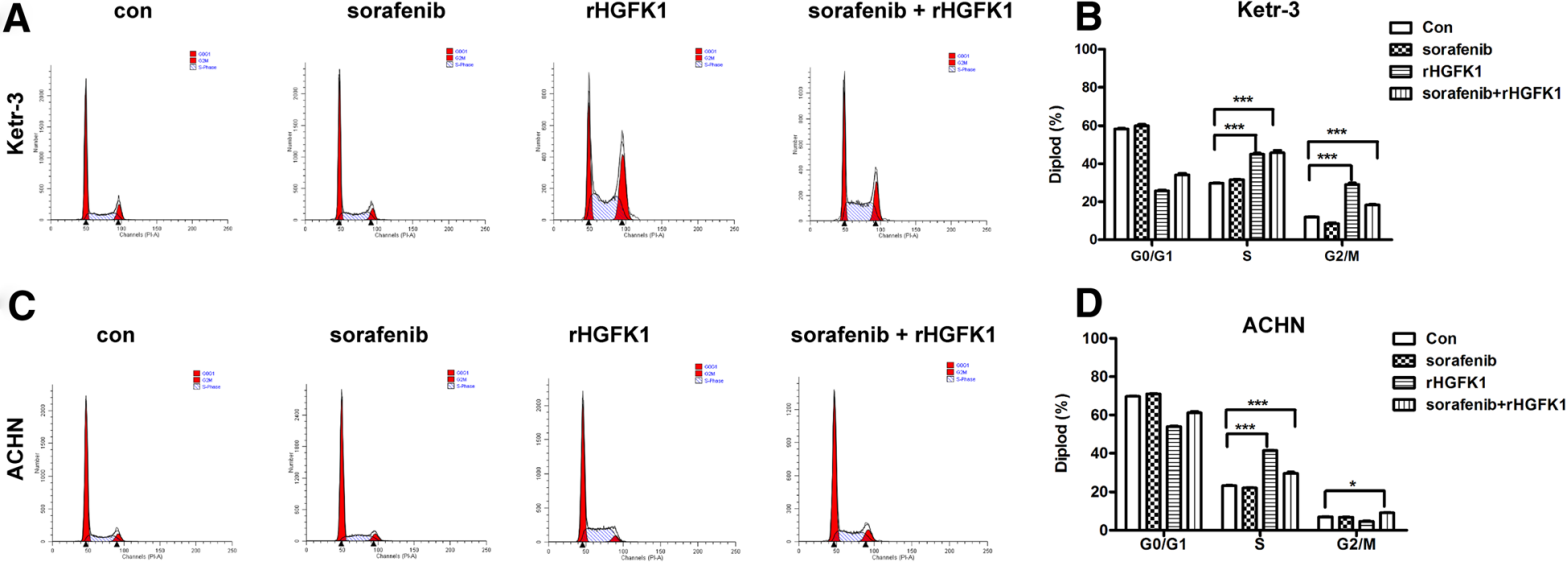
Effect of sorafenib and HGFK1 on cell cycle arrest of RCC. Ketr-3 (**a**) and ACHN (**c**) cells were treated with sorafenib and/or rHGFK1 for 48 h and stained with PI, and then analyzed with a flow cytometer. Quantification of cell cycle arrest is shown for Ketr-3 (**b**) and ACHN (**d**) induced by sorafenib and/or rHGFK1. All data shown represent the mean ± SD from at least three independent experiments. Significant differences are denoted by * for *p* < 0.05, and *** for *p* < 0.001

We further investigated the effects of sorafenib and HGFK1 on cell cycle arrest of RCC. Ketr-3 and ACHN cells were treated with sorafenib and/or rHGFK1, and then analyzed cell cycle with flow cytometry. As shown in Fig. 5a–d, sorafenib treatment (6.0 μmol/l) did not affect the cell cycle on Ketr-3 and ACHN cells. However, rHGFK1 treatment (2.0 μmol/l) could significantly induce S and G2/M-phase cell cycle arrest on Ketr-3 cells, and S-phase cell cycle arrest on ACHN cells. Moreover, combination treatment of rHGFK1 and sorafenib could significantly induce S and G2/M-phase cell cycle arrest on Ketr-3 and ACHN cells.

### Sorafenib induces autophagy to promote cell survival in RCC

**Fig. 6:**
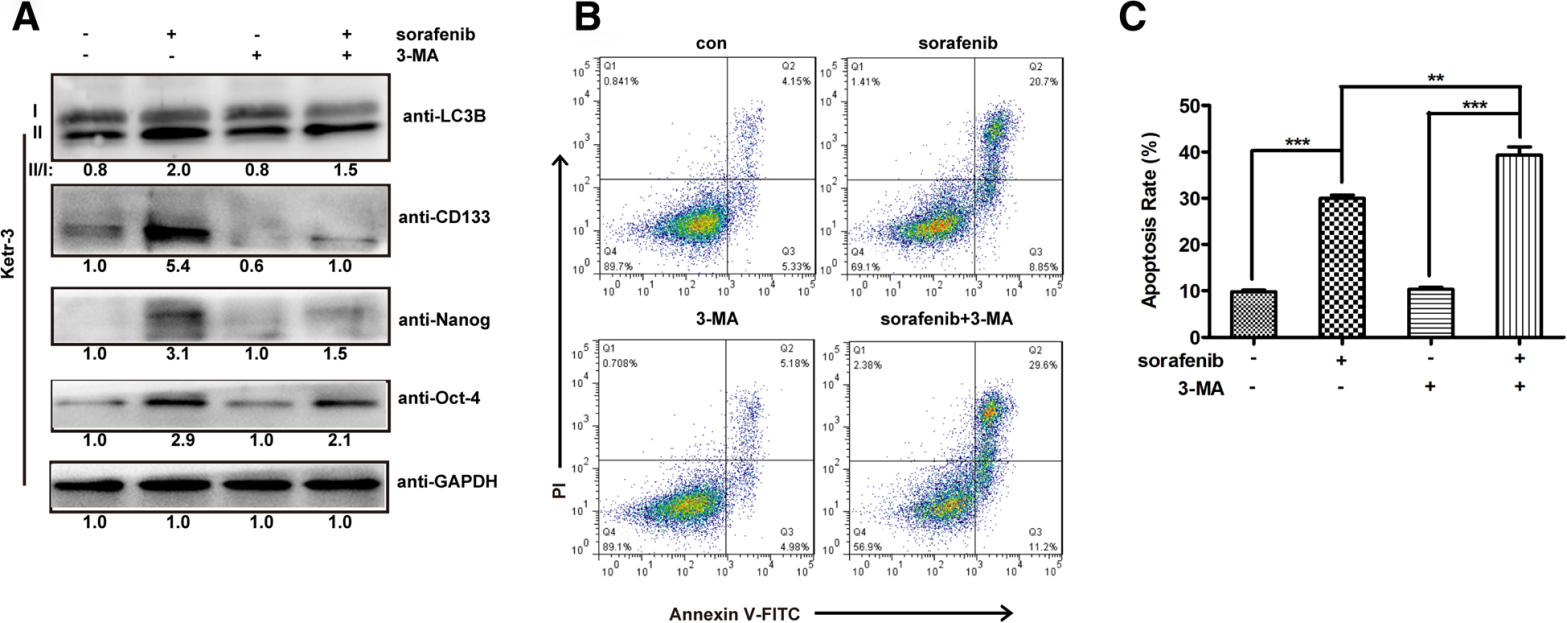
Sorafenib-induced autophagy promotes RCC cell survival. Ketr-3 cells were treated with sorafenib and/or 3-MA for 48 h, and then analyzed the associated protein expression with Western-Blotting (**a**) and the cell apoptosis with an Annexin V-FITC/PI apoptosis kit (**b**). **c** Quantification of cell apoptosis is shown for Ketr-3 induced by sorafenib and/or 3-MA. All data shown represent the mean ± SD from at least three independent experiments. Significant differences are denoted by ** for *p* < 0.01, and *** for *p* < 0.001

Autophagy is an important intracellular catabolic process for maintaining cellular homeostasis via degrading misfolded proteins or damaged organelles [31]. In our present study, sorafenib treatment could promote the conversion of cytosolic LC3B-I to autophagosome-associated LC3B-II (a well-known marker for cellular autophagy) in Ketr-3 cells, which suggested that sorafenib treatment could induce autophagy (Fig. 6a). 3-MA, an inhibitor of autophagy, combined treatment with sorafenib could inhibit the up-regulation of LC3B-II induced by sorafenib (Fig. 6a). In order to further explore whether autophagy induced by sorafenib could result in cell apoptosis or rescue cell survival of RCC, the apoptosis of Ketr-3 cells treated with sorafenib and/or 3-MA was analyzed by using flow cytometry. As shown in Fig. 6b and c, sorafenib treatment could induce cell apoptosis (29.96%), but 3-MA treatment did not cause obvious cell apoptosis compared to the control. Surprisingly, combination treatment of sorafenib and 3-MA could induce higher cell apoptosis (39.3%) than anyone of them. Our results implied that sorafenib could induce autophagy to promote cell survival in RCC.

### Sorafenib induces autophagy to enhance cancer stemness of RCC

Increasing evidences suggested that sustained activation of autophagy was associated with the maintenance of cancer stem cells (CSCs)-like phenotype that is an important cause for cancer drug-resistance [32]. CD133, a surface expressed protein, is considered as one of the most important CSCs markers in variety of solid tumors, including colorectal cancer, HCC, pancreatic cancer, and RCC [33]. Moreover, Nanog and Oct-4 have also been proved to play important roles in stem cell self-renewal as stem cell associated transcriptional factors [34, 35]. Here, our data demonstrated that sorafenib treatment could induce the expression of CD133, Nanog, and Oct-4 in Ketr-3 cells (Fig. 6a). Besides, 3-MA not only inhibited autophagy but also down-regulated the expression of CD133, Oct-4, and Nanog induced by sorafenib (Fig. 6a). So those results implied that sorafenib-induced autophagy was contributed to improve cancer stemness of RCC.

### HGFK1 inhibits sorafenib-induced autophagy via inhibiting NF-κB pathway in RCC

**Fig. 7:**
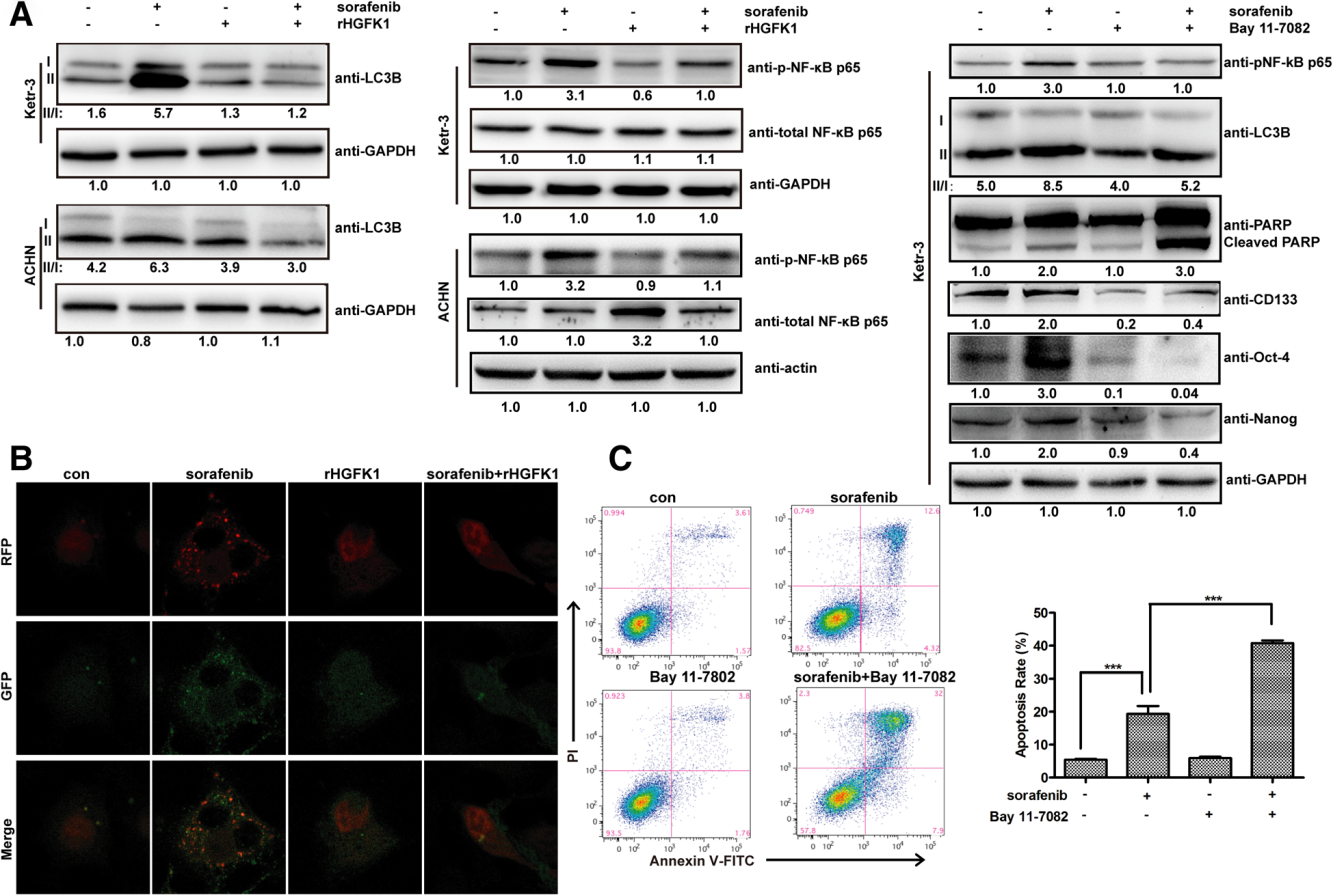
HGFK1 inhibits sorafenib-induced autophagy via inhibiting NF-κB signaling pahtway. **a** Ketr-3 and ACHN cells were treated with sorafenib and/or rHGFK1, Bay 11–7082, then analyzed by Western-Blotting. **b** Ketr-3 cells were treated with sorafenib and/or rHGFK1, then analyzed by mRFP-GFP-LC3 puncta formation assay. **c** Ketr-3 cells were treated with sorafenib and/or Bay 11–7082, then analyzed the cell apoptosis by flow cytometer. Significant differences are denoted by *** for *p* < 0.001

HGFK1, as a potent anti-angiogenic factor, exhibits anti-tumor activity on breast cancer [20], colon cancer [21], glioblastoma [24], and HCC [22]. Interestingly, combination treatment of rHGFK1 and sorafenib could inhibit the increase of LC3B-II induced by sorafenib in Ketr-3 and ACHN cells (Fig. 7a). In order to further confirm that HGFK1 can inhibit sorafenib-induced autophagy, autophagy flux was detected by mRFP-GFP-LC3 puncta formation assay in Ketr-3 cells. As shown in Fig. 7b, sorafenib could induce puncta formation (corresponding to yellow autophagosome and red autolysosomes) but not rHGFK1, and combination of sorafenib and rHGFK1 inhibited puncta formation that further demonstrated that HGFK1 could inhibit sorafenib-induced autophagy.

It has also been reported that activation of NF-κB signaling pathway can trigger autophagy after heat shock and hypoxia [36]. NF-κB inhibition could enhance the sensitivity of HCC cells to sorafenib [37]. As shown in Fig. 7a, sorafenib treatment induced phosphorylation of NF-κB p65 at Serine-311 in Ketr-3 and ACHN cells. And HGFK1 treatment could inhibit the phosphorylation of NF-κB p65 induced by sorafenib, which implied that HGFK1 could inhibit sorafenib-induced autophagy via suppressing NF-κB signaling pathway.

In order to further clarify the role of NF-κB activation in the anti-tumor activity of sorafenib in RCC, Bay 11–7082 (inhibitor of NF-κB, which decreases NF-κB by inhibiting TNF-α-induced phosphorylation of IκB-α) was used for combination with sorafenib in Ketr-3 cells. As shown in Fig. 7c, sorafenib treatment induced 19.34% cell apoptosis, Bay 11–7082 did not induce obvious cell apoptosis, but combination of Bay 11–7082 and sorafenib induced 40.76% cell apoptosis. Western-Blotting assay results showed that combination of Bay 11–7082 and sorafenib could result in more cleavage of PARP compared to sorafenib treatment. Additionally, Bay 11–7082 treatment also down-regulated the expression of LC3B-II, CD133, Oct-4 and Nanog, which suggested that inhibition of NF-κB activation could inhibit autophagy and impair the stemness in RCC.

### HGFK1 suppresses sorafenib-induced cancer stemness in RCC

**Fig. 8:**
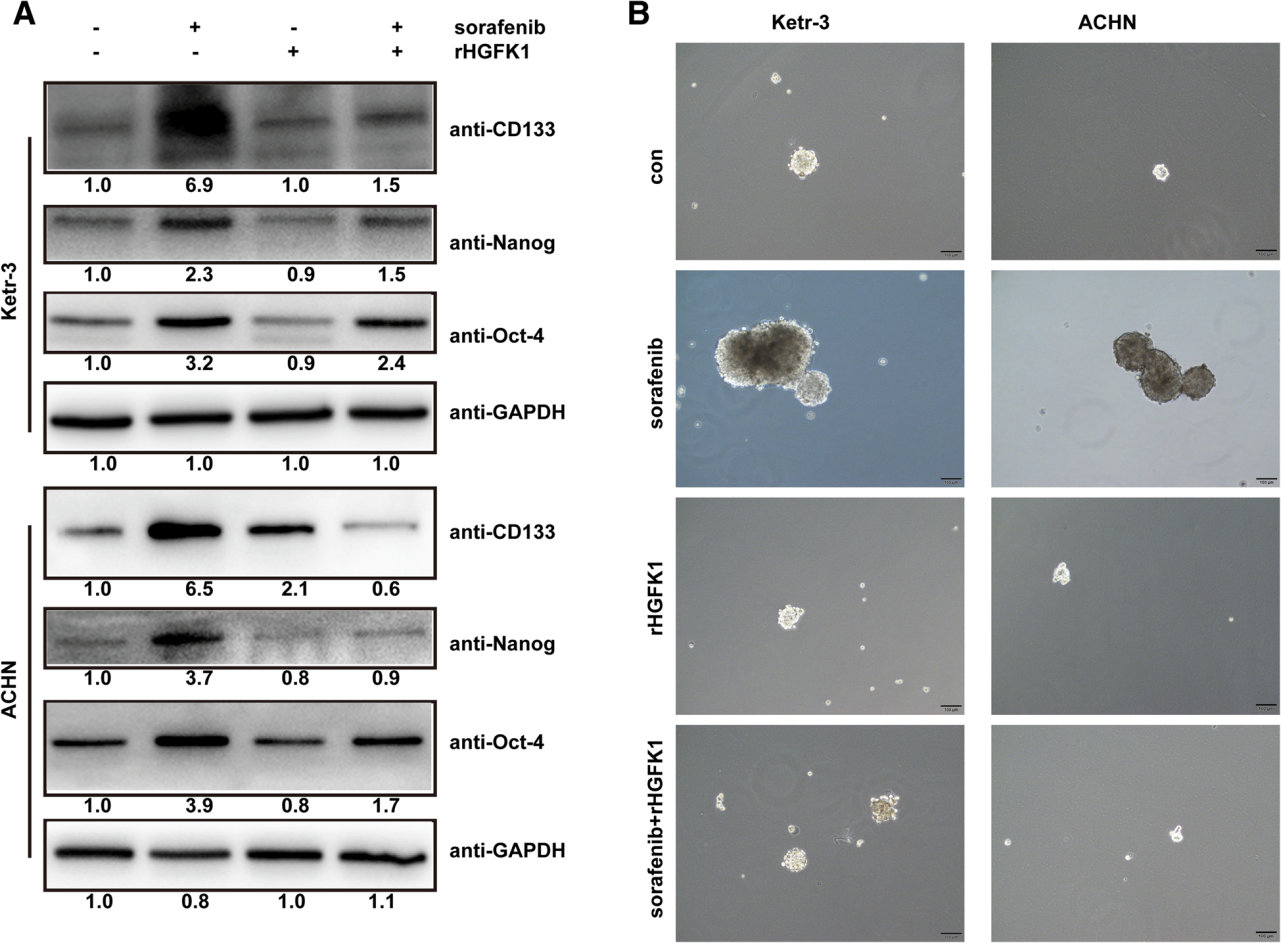
HGFK1 suppresses sorafenib-induced cancer stemness in RCC. **a** Ketr-3 and ACHN cells were treated with sorafenib and/or rHGFK1 for 48 h, then analyzed the expression of CD133, Nanog, and Oct4 with Western-Blotting. **b** Mammosphere formation assay was used to analyze the cancer stemness of Ketr-3 and ACHN cells after treated with sorafenib and/or rHGFK1

In order to investigate whether HGFK1 could suppress cancer stemness of RCC via inhibiting sorafenib-induced autophagy, combination treatment of rHGFK1 and sorafenib was performed in Ketr-3 and ACHN cells. Our results showed that rHGFK1 rarely effected the expression of CD133, Oct-4, and Nanog, but it could significantly decrease the up-regulation of CD133, Oct4, and Nanog induced by sorafenib (Fig. 8a). Mammosphere formation assay results showed that sorafenib not rHGFK1 treatment enhanced the mammosphere formation compared to the control. And combination of rHGFK1 and sorafenib reduced the mammosphere formation (Fig. 8b). sorafenib can inhibit cell proliferation of RCC, but it could also increase the stemness properties of RCC leading to drug resistance, which discounts its anti-tumor activity. Our finds showed that HGFK1 was able to inhibit sorafenib-induced cancer stemness of RCC, which implied that combination of HGFK1 and sorafenib might exhibit synergistic anti-tumor activity in RCC.

### HGFK1 inhibits autophagy and cancer stemness of RCC via down-regulation of NF-κB activation induced by sorafenib in vivo

**Fig. 9:**
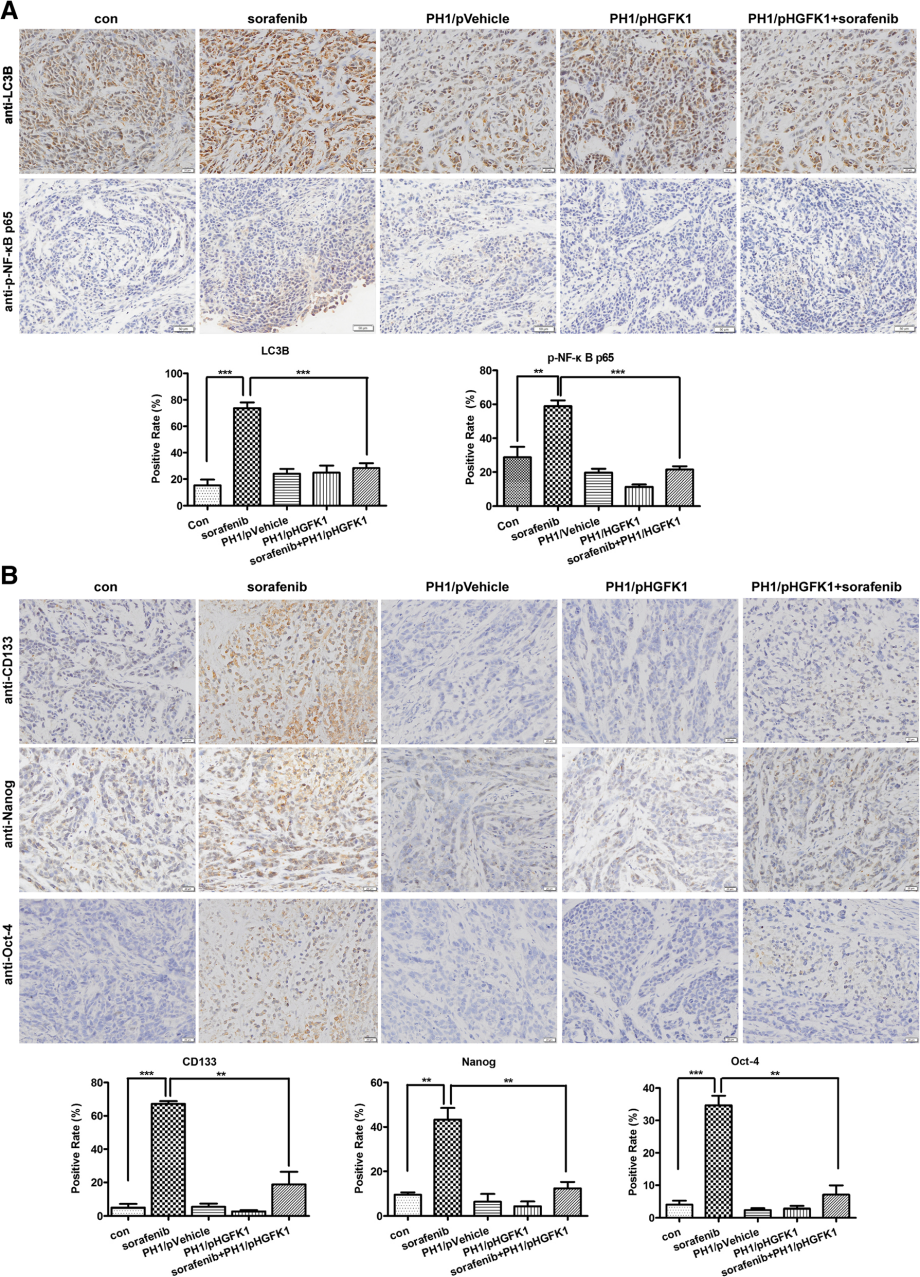
Effect of sorafenib and HGFK1 on the expression of LC3B, phosphorylated NF-κB, CD133, Nanog, and Oct4 in vivo. Tumor tissues from sorafenib and/or HGFK1 treated tumor-bearing mice were collected and stained with IHC. The expression of LC3B and phosphorylated NF-κB (**a**), as well as CD133, Nanog, and Oct4 (**b**) on tumor tissues was analyzed and quantified with IOD. All data shown represent the mean ± SD from three to five independent sections. Significant differences are denoted by ** for *p* < 0.01, and *** for *p* < 0.001

To explore whether HGFK1 and sorafenib affect autophagy and presence of CSCs of RCC in vivo, the expression of LC3B, phosphorylated NF-κB**,** and CSCs markers were analyzed with IHC staining on xenograft tumor as described in *materials* and *methods*. Compared to the controls, sorafenib could increase LC3B expression but not HGFK1; however, the up-regulation of LC3B induced by sorafenib could be inhibited by HGFK1. In addition, sorafenib treatment induced significant activation of NF-κB, which is consistent with the in vitro results (Fig. 9a). Moreover, sorafenib could up-regulate the expression of CD133, Nanog, and Oct-4; surprisingly, these expressions could also be decreased by HGFK1 (Fig. 9b). Taken together, our results further demonstrated that HGFK1 could inhibit the activation of autophagy and prevent the increasing of CSCs subpopulation induced by sorafenib in RCC xenogfrat mouse model.

## Discussion

sorafenib, an oral multikinase inhibitor, is still recommended as one of the first-line therapy for patients with advanced RCC [38]. However, drug resistance is frequently observed in RCC patients after sorafenib treatment [39]. Drug resistance of sorafenib can be cataloged into two types including primary/intrinsic resistance and secondary/acquired one [40]. However, the mechanisms of sorafenib resistance are still complex and unclarified. CSCs, a small subpopulation within tumors, have self-renewal capacity and multipotency, and play a critical role in tumorigenesis, metastasis, tumor recurrence, as well as drug resistance [41], which has also been defined as the culprit of primary drug resistance in many malignances [42]. In addition, undergoing the selective pressures originated from insufficient dose of chemotherapy and radiation therapy, cancer cells can hijack stemness properties of CSCs to adapt the harsh environment and avoid to be eradicated [43, 44]. It has been reported that sorafenib-induced drug resistance and cancer recurrence are associated with the enhanced stemness properties of cancer cells in HCC [13, 15, 40]. And inhibition of CSCs or stemness-high cancer cells could significantly suppress cancer relapse and metastasis [45]. Accordingly, cancer stemness property is a reasonable target for reversing acquired drug resistance of cancer cells. However, there is no evidence to prove the regulation of sorafenib on cancer stemness of RCC.

Autophagy, a self-degradation process that is required for maintaining cellular homeostasis, is widely implicated in many pathophysiological diseases, including cancer [46]. Initially, autophagy is proposed as a tumor suppresser because of its deficiency causing oxidative stress, DNA damage, and genome instability that are known associated with tumorigenesis in some context [47]. As now, considerable evidences supported that up-regulation of autophagy could promote cancer cell survival and drug-resistance to adapt microenvironment stresses such as starvation, hypoxia, chemotherapy, and radiation therapy [47, 48]. Thus, the role of autophagy in cancer is complex and context dependent. In our present study, the surviving RCC cells, which have been treated with sorafenib at the concentration of IC50, exhibited significant activation of autophagy (Fig. 6). Interestingly, autophagy is considered as survival pathway for cancer cells responding to stress condition and also involved in the maintenance of stem-like states of embryonic stem cells, tissue stem cells, and CSCs [46, 49]. Here, our data showed that sorafenib-induced autophagy was involved in the up-regulation of stemness associated genes, including CD133, Nanog, and Oct4 (Fig. 6). These results further indicate that the stemness properties of RCC cells can be enhanced by the insufficient lethal dose of sorafenib treatment via activating autophagy, which acts as a preserving mechanism and contributes to the evolution of sorafenib acquired resistance.

HGF is a paracrine cytokine associated with cellular growth, motility, and morphology. c-MET (cellular mesenchymal–epithelial transition factor) is the unique receptor of HGF with kinase activity [50]. Binding of HGF can induce c-MET dimerization, also activate its autophosphorylation and downstream signaling pathways including ERK1/2, PI3K, NF-κB and STAT3/5 [51]. Increasing evidence demonstrates that abnormal activation of HGF/c-MET was involved in the cancer progression. Thus, HGF/c-MET is an important cancer therapeutic target [52]. According to our recent results, HGFK1 could inhibit c-MET activity in vitro and in vivo acting as its inhibitor [24]. As previously reported, HGFK1 could interfere the activities of EGF/EGFR and bFGF/bFGFR singling pathways in HCC [22]. Interestingly, these pathways are the most important regulators of human embryonic stem cell self-renewal and cancer cell tumorigenesis [53, 54]. In addition, EGF and bFGF can also be used to culture the CSCs of RCC as supplements in vitro and play critical roles in maintaining self-renewal ability of RCC [55]. Thus, HGFK1 is able to block EGF and bFGF signaling to inhibit stemness of RCC. Interestingly, our study figured out that HGFK1 could reverse sorafenib-induced stemness of RCC via inhibiting autophagy, and exhibit synergistic anti-tumor activity on RCC both in vitro and in vivo. Furthermore, our results showed that HGFK1 could inhibit sorafenib-induced autophagy via blockading NF-κB signaling pathway, which should also conduce to inhibit stemness of RCC.

For a long time, a safe and effective drug delivery technique is required for in vivo study of a therapeutic peptide. In our study, polymeric nanoparticles formed by plasmids encoding the secreted HGFK1 protein (PH1/pHGFK1) was chosen to continuously and effectively supply HGFK1 peptide. Importantly, PH1 is a biodegradable, high efficacy and low toxicity gene delivery vector (Fig. 1). In addition, intravenous administration can improve the systemic circulation and decrease the degradation by digestive system of nanoparticles. Our in vivo studies showed that tail vein injection of PH1/pHGFK1 nanoparticles could significantly inhibit tumor growth and prolong the survival time of tumor-bearing mice in the xenograft tumor mice model (Fig. 2), which should be attributed to the continuous and effective releasing of HGFK1 by tissues and cells transfected with PH1/pHGFK1. Particularly, consisting with the observations of our animal studies, PH1/pHGFK1 treatment could significantly reverse the stemness gene expression of CD133, Nanog, and Oct4 in tumor tissues induced by sorafenib. Moreover, combination treatment of PH1/pHGFK1 and sorafenib could also enhance the anti-tumoral efficacies via repressing tumor growth and stemness genes expression (Figs. 2 and 9). To summarize, PH1/pHGFK1 could synergistically improve anti-tumoral functions of sorafenib in vivo.

## Conclusion

**Fig. 10:**
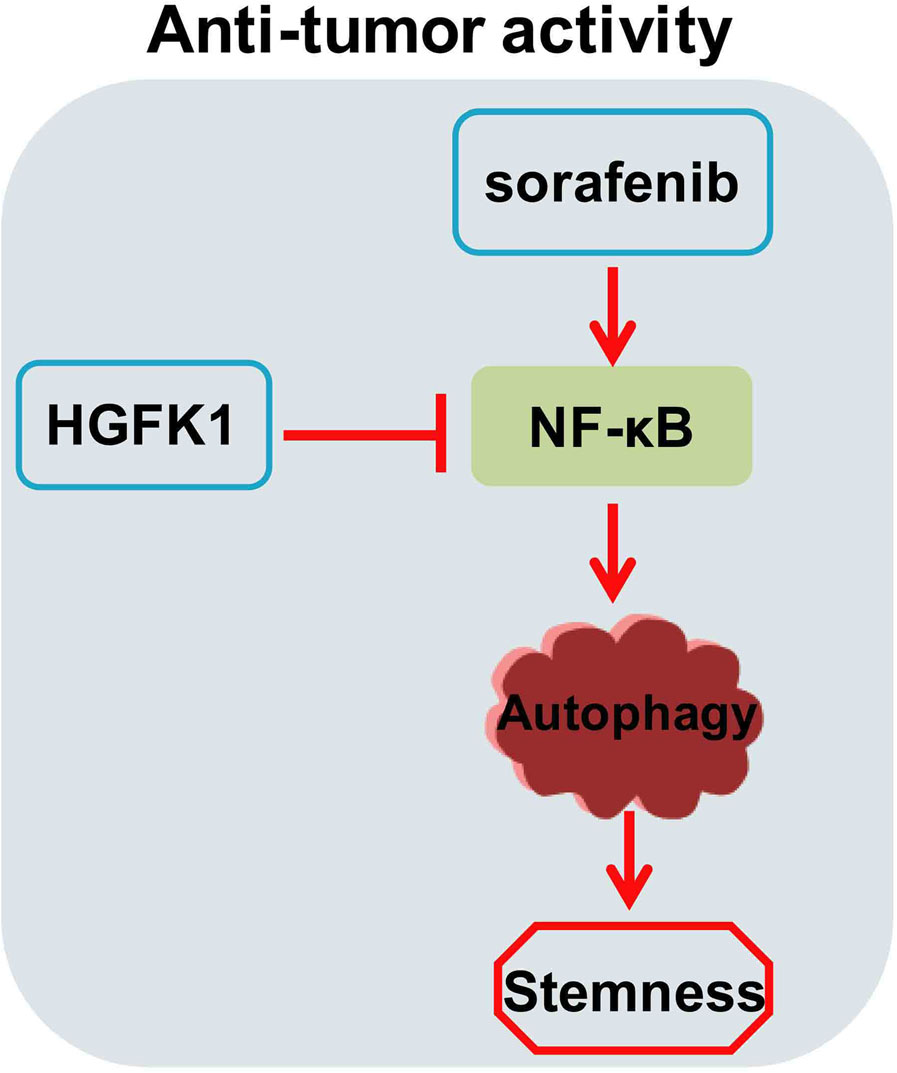
Schematic illustration of HGFK1 and sorafenib exhibiting synergistic anti-tumor effects on RCC. In brief, sorafenib could induce autophagy via activating NF-κB signaling pathway, and then enhance the cancer stemness of RCC resulting in drug resistance. But HGFK1 could inhibit the activation of NF-κB induced by sorafenib

HGFK1 exhibits anti-tumor activity, and synergistically enhances the anti-tumor activity of sorafenib on RCC both in vitro and in vivo. Furthermore, HGFK1 is able to inhibit sorafenib-induced autophagy via blockading NF-κB signaling pathway, which is responsible for suppressing tumor growth and stemness of RCC (Fig. 10). Taken together, our results provide rational basis for clinical application of sorafenib and HGFK1 combination therapy in RCC patients.

## Additional file

Additional file 1: **Figure S1.** Distribution and circulation analysis of PH1/pVehicle nanoparticles was performed with *in vivo* optical imaging systems. The tumor-bearing mice were given PH1 loaded control plasmids (PH1/pVehicle, which expresses GFP protein) by intravenous injection. The GFP fluorescence was monitored via *in vivo* optical imaging system at the indicated time points. **Figure S2.** The body weight of tumor-bearing mice. The body weight of tumor-bearing mice was recorded every three days, all data shown represent the mean ± SD from ten independent mice. No significant differences were presented in the body weight between differential treatment and control tumor-bearing mice. **Figure S3.** HE staining of internal organs for tumor-bearing mice. Following treatment for 28 days, the internal organs including lung, heart, liver, spleen, and kidney from the tumor-bearing mice were HE stained. No differences were seen in the organs between differential treatment and control tumor-bearing mice. **Figure S4.** Preparation of recombinant HGFK1 protein. The fusion protein containing recombinant HGFK1 and intein tag, which was expressed in *E. coli* BL21 (DE3), were purified using chitin affinity beads and then cleaved using DTT. The purified rHGFK1 produced a single 11 kDa band. (DOC 20027 kb)

## Data Availability

All data generated or analyzed during this study are included in this published article and its supplementary information files.

## Abbreviations

c-MET: Cellular mesenchymal–epithelial transition factor
CSCs: Cancer stem cells
HCC: Hepatocellular carcinoma
HGF: Hepatocyte growth factor
HGFK1: Kringle 1 domain of human hepatocyte growth factor
PEG: Polyethylene glycol
PH1: Peglated-H1
RCC: Renal cell carcinoma
RES: Reticuloendothelial system
USFDA: United States Food and Drug Administration
