# Correction: Peglated-H1/pHGFK1 nanoparticles enhance anti-tumor effects of sorafenib by inhibition of drug-induced autophagy and stemness in renal cell carcinoma

**DOI:** 10.1186/s13046-023-02669-2

**Published:** 2023-04-18

**Authors:** Xiaoge Gao, Pin Jiang, Qian Zhang, Qian Liu, Shuangshuang Jiang, Ling Liu, Maomao Guo, Qian Cheng, Junnian Zheng, Hong Yao

**Affiliations:** 1 Cancer Institute, Xuzhou Medical University, Xuzhou, Jiangsu Province 221002 People’s Republic of China; 2 Center of Clinical Oncology, Affiliated Hospital of Xuzhou Medical University, Xuzhou, Jiangsu Province 221002 People’s Republic of China; 3 Department of Cancer Biotherapy Center, Third Affiliated Hospital of Kunming Medical University, Kunming, Yunnan Province 650118 People’s Republic of China

**Correction** **:** **J Exp Clin Cancer Res 38, 362 (2019)**

**https://doi.org/10.1186/s13046-019-1348-z**

Following publication of the original article [1], the authors identified an error in Fig. 9, specifically:

- Fig. 9a - the representative IHC figure of LC3B in PH1/HGFK1 + sorefenib group was repetitive to PH1/pVehicle group

The correct figure is given below.

**Fig. 9:**
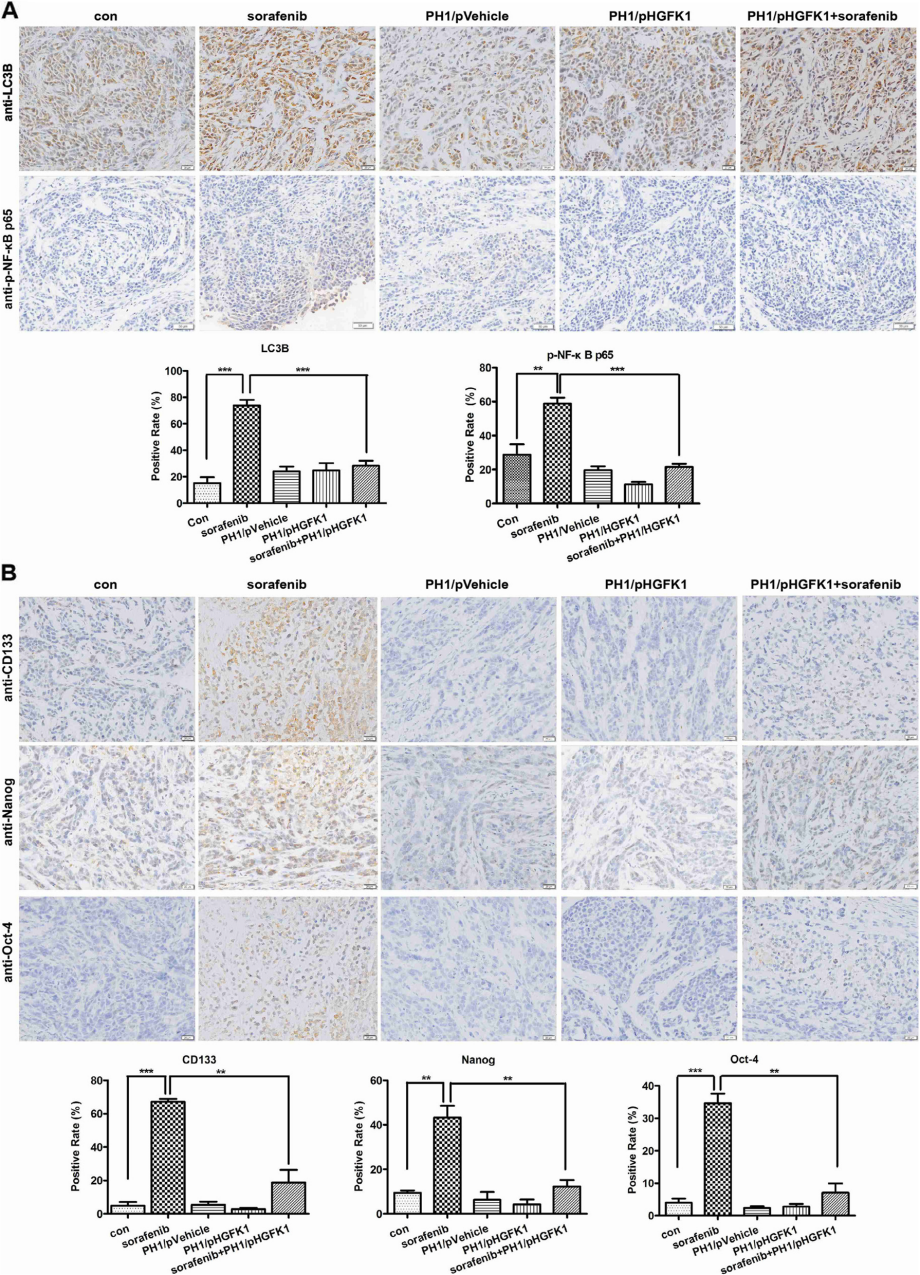
Effect of sorafenib and HGFK1 on the expression of LC3B, phosphorylated NF-κB, CD133, Nanog, and Oct4 in vivo. Tumor tissues from sorafenib and/or HGFK1 treated tumor-bearing mice were collected and stained with IHC. The expression of LC3B and phosphorylated NF-κB (**a**), as well as CD133, Nanog, and Oct4 (**b**) on tumor tissues was analyzed and quantified with IOD. All data shown represent the mean ± SD from three to five independent sections. Significant differences are denoted by ** for *p* < 0.01, and *** for *p* < 0.001
