# Supplementary material for: Peglated-H1/pHGFK1 nanoparticles enhance anti-tumor effects of sorafenib by inhibition of drug-induced autophagy and stemness in renal cell carcinoma
Source: J Exp Clin Cancer Res. 2019 Aug 19;38:362. doi: 10.1186/s13046-019-1348-z (PMC6699135; doi:10.1186/s13046-019-1348-z)
Supplement: Supplementary file 1 — Figure S1. Distribution and circulation analysis of PH1/pVehicle nanoparticles was performed with in vivo optical imaging systems. The tumor-bearing mice were given PH1 loaded control plasmids (PH1/pVehicle, which expresses GFP protein) by intravenous injection. The GFP fluorescence was monitored via in vivo optical imaging system at the indicated time points. Figure S2. The body weight of tumor-bearing mice. The body weight of tumor-bearing mice was recorded every three days, all data shown represent the mean ± SD from ten independent mice. No significant differences were presented in the body weight between differential treatment and control tumor-bearing mice. Figure S3. HE staining of internal organs for tumor-bearing mice. Following treatment for 28 days, the internal organs including lung, heart, liver, spleen, and kidney from the tumor-bearing mice were HE stained. No differences were seen in the organs between differential treatment and control tumor-bearing mice. Figure S4. Preparation of recombinant HGFK1 protein. The fusion protein containing recombinant HGFK1 and intein tag, which was expressed in E. coli BL21 (DE3), were purified using chitin affinity beads and then cleaved using DTT. The purified rHGFK1 produced a single 11 kDa band. (DOC 20027 kb) [file 13046_2019_1348_MOESM1_ESM.doc]

**Title:** Peglated-H1/pHGFK1 nanoparticles enhance anti-tumor effects of sorafenib by inhibition of drug-induced autophagy and stemness in renal cell carcinoma

**Running title:** HGFK1 enhances anti-tumor effects of sorafenib in RCC

Xiaoge Gao^1, 2#^, Pin Jiang^1, 2#^, Qian Zhang^1, 2^, Qian Liu^1^, Shuangshuang Jiang^1^, Ling Liu^1^, Maomao Guo^1, 2^, Qian Cheng^1, 2^, Junnian Zheng^1, 2*^ and Hong Yao^1, 2*^

1. Cancer Institute, Xuzhou Medical University, Xuzhou, Jiangsu Province, 221002, P. R. China

2. Center of Clinical Oncology, Affiliated Hospital of Xuzhou Medical University, Xuzhou, Jiangsu Province, 221002, P. R. China

3. Department of Cancer Biotherapy Center, Third Affiliated Hospital of Kunming Medical University, Kunming, Yunnan Province, 650118, P. R. China

^#^ means equal contribution to this study.

* Correspondence to: Prof. Hong Yao, E-mail address: [yaohong20055@hotmail.com](mailto:yaohong20055@hotmail.com6-85582530). Prof. Junnian Zheng, E-mail address: [jnzheng@xzhmu.edu.cn](mailto:jnzheng@xzhmu.edu.cn).

Xiaoge Gao, E-mail: [xiaogegao@xzhmu.edu.cn](mailto:xiaogegao@xzhmu.edu.cn)

Pin Jiang, E-mail: [1045581887@qq.com](mailto:1045581887@qq.com)

Qian Zhang, E-mail: [513408561@qq.com](mailto:513408561@qq.com)

Qian Liu, E-mail: [776593900@qq.com](mailto:776593900@qq.com)

Shuangshuang Jiang, E-mail: [286785928@qq.com](mailto:286785928@qq.com)

Ling Liu, E-mail: [liulhyn@126.com](mailto:liulhyn@126.com)

Maomao Guo, E-mail: [353591556@qq.com](mailto:353591556@qq.com)

Qian Cheng, E-mail: [xss023@126.com](mailto:xss023@126.com)

Junnian Zheng, E-mail: [jnzheng@xzhmu.edu.cn](mailto:jnzheng@xzhmu.edu.cn)

Hong Yao, E-mail: yaohong20055@hotmail.com

**Figure S1. Distribution and circulation analysis of PH1/pVehicle nanoparticles was performed with *in vivo* optical imaging systems.** The tumor-bearing mice were given PH1 loaded control plasmids (PH1/pVehicle, which expresses GFP protein) by intravenous injection. The GFP fluorescence was monitored via *in vivo* optical imaging system at the indicated time points.

**
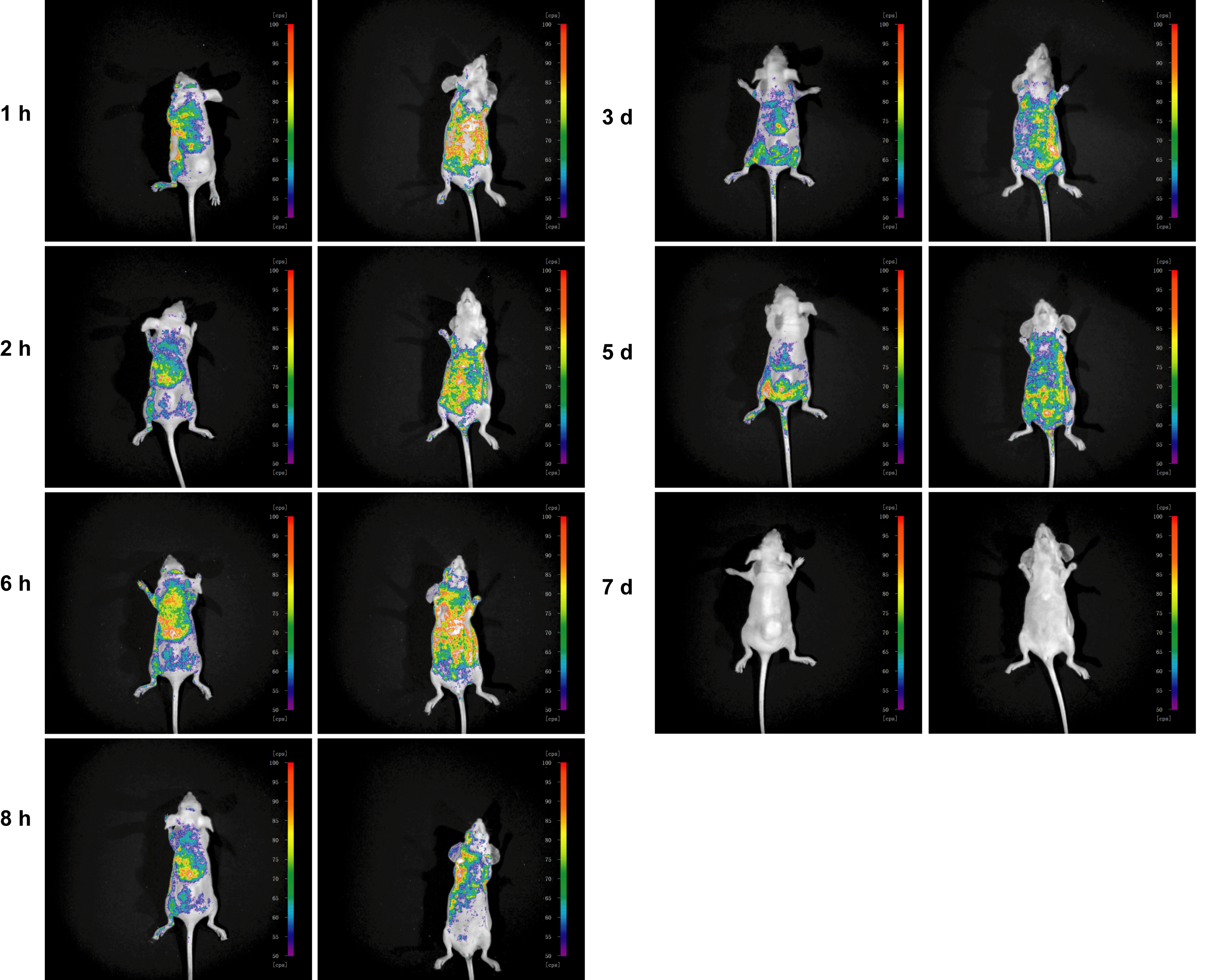
**

**Figure S2. The body weight of tumor-bearing mice**. The body weight of tumor-bearing mice was recorded every three days, all data shown represent the mean ± SD from ten independent mice. No significant differences were presented in the body weight between differential treatment and control tumor-bearing mice.


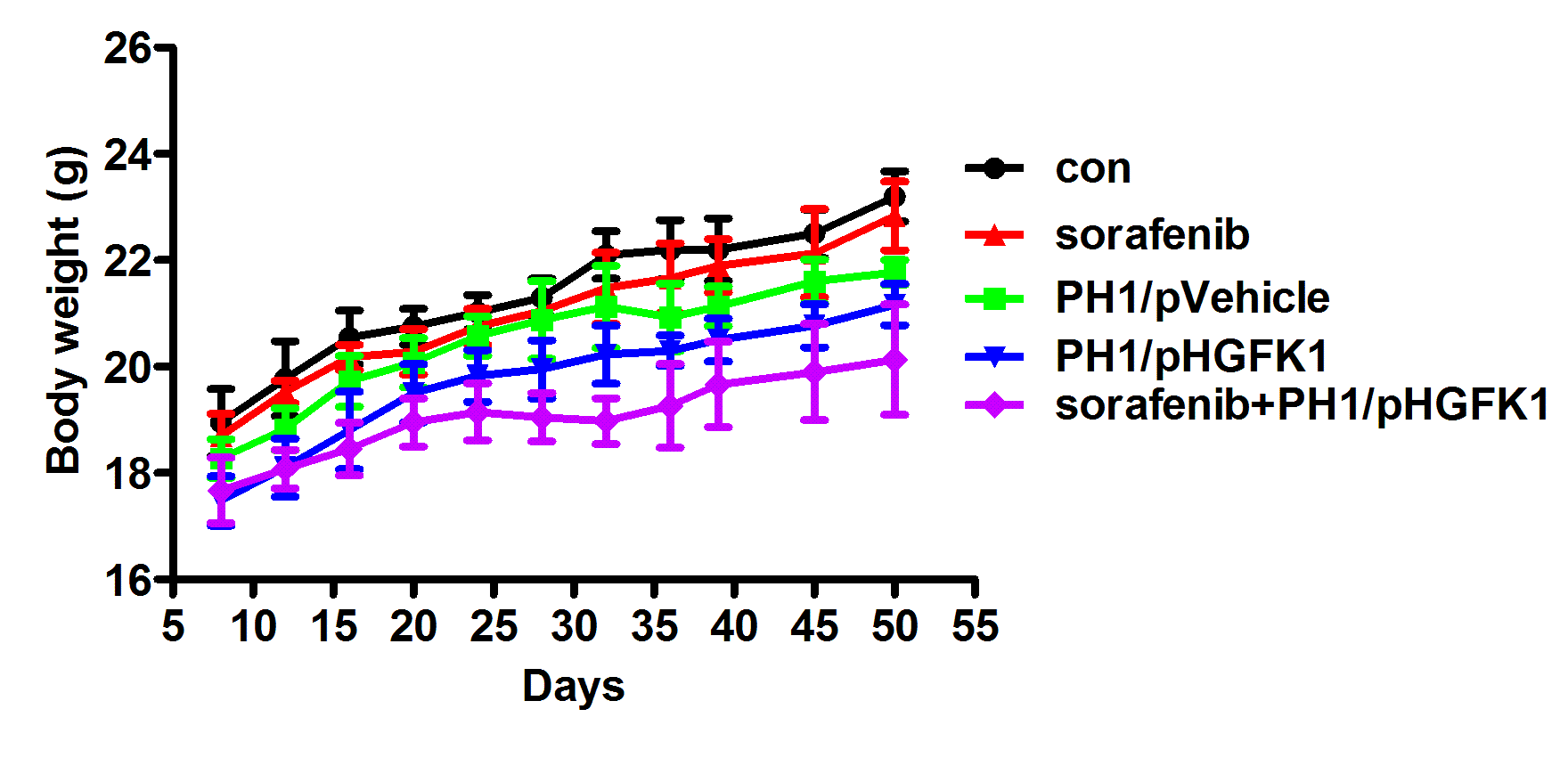


**Figure S3. HE staining of internal organs for tumor-bearing mice.** Following treatment for 28 days, the internal organs including lung, heart, liver, spleen, and kidney from the tumor-bearing mice were HE stained. No differences were seen in the organs between differential treatment and control tumor-bearing mice.

**
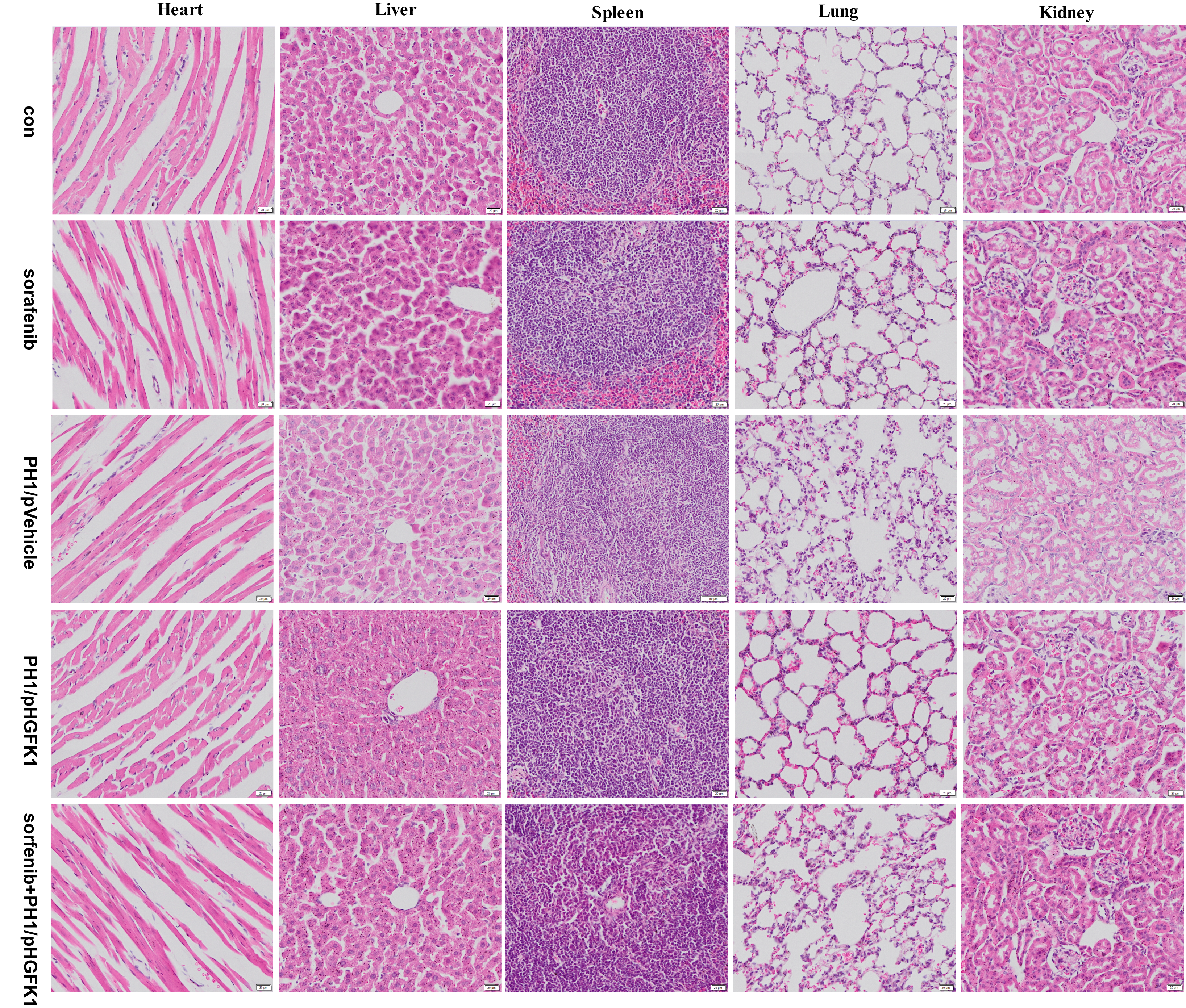
**

**Figure S4. Preparation of recombinant HGFK1 protein.** The fusion protein containing recombinant HGFK1 and intein tag, which was expressed in *E. coli* BL21 (DE3), were purified using chitin affinity beads and then cleaved using DTT. The purified rHGFK1 produced a single 11 kDa band.

**
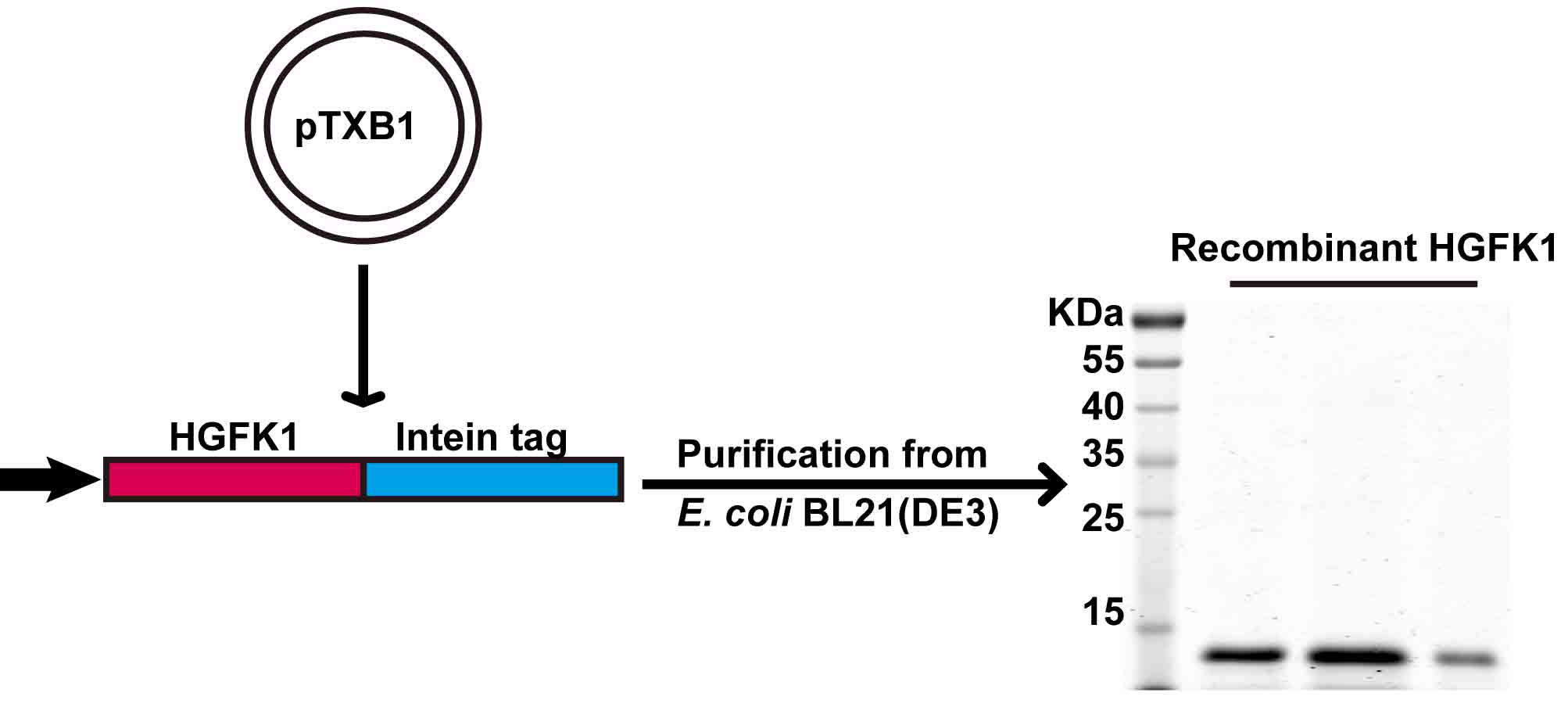
**
